# Oral ketone monoester supplementation does not accelerate recovery of muscle force or modulate circulating cytokine concentrations after muscle‐damaging eccentric exercise in healthy males and females

**DOI:** 10.1113/EP090546

**Published:** 2022-09-26

**Authors:** Tom S. O. Jameson, Hashim Islam, Benjamin T. Wall, Jonathan P. Little, Francis B. Stephens

**Affiliations:** ^1^ Nutritional Physiology Group Department of Sport and Health Sciences College of Life and Environmental Sciences University of Exeter Exeter UK; ^2^ School of Health and Exercise Sciences University of British Columbia Okanagan Campus Kelowna BC Canada

**Keywords:** β‐Hydroxybutyrate, BOHB, cytokine, inflammation, TRAIL

## Abstract

**New Findings:**

- **What is the central question of this study?**
  Does acute ketone monoester supplementation enhance the recovery of muscle force and modulate circulating cytokine concentrations after muscle‐damaging eccentric exercise?
- **What is the main finding and its importance?**
  Ketone monoester supplementation increased plasma β‐hydroxybutyrate concentrations but did not attenuate the reduction in muscle force or the increase in plasma inflammatory cytokine concentrations that occurred after eccentric exercise. Notably we report novel data demonstrating a reduction in plasma TRAIL concentrations after eccentric exercise, highlighting TRAIL signalling as a possibly novel regulator of muscle recovery.

**Abstract:**

Muscle‐damaging eccentric exercise is associated with inflammation and impaired muscle force. β‐Hydroxybutyrate (β‐OHB) reduces muscle protein breakdown during inflammation but whether oral ketone monoester supplementation accelerates recovery of muscle force after eccentric exercise is unknown. Sixteen healthy males and females consumed thrice daily ketone monoester (27 g per dose; *n* = 8; six females; KES) or isocaloric maltodextrin placebo (*n* = 8; four females; PLA) drinks (randomized, double‐blind, parallel group design) for 3 days beginning immediately after 300 unilateral eccentric quadriceps contractions during complete eucaloric dietary control (1.2 ± 0.1 g/kg BM/day standardized protein). Bilateral muscle force measurements and venous blood sampling were performed before and 3, 6, 24, 48 and 72 h after eccentric exercise. Plasma β‐OHB concentrations were greater in KES compared with PLA at 3 h (0.56 ± 0.13 *vs*. 0.22 ± 0.04 mM, respectively; *P* = 0.080) and 6 h (0.65 ± 0.41 *vs*. 0.23 ± 0.02 mM, respectively; *P* = 0.031) post‐eccentric exercise. Relative to the control leg, isokinetic work (by 20 ± 21% in PLA and 21 ± 19% in KES; *P* = 0.008) and isometric torque (by 23 ± 13% in PLA and 20 ± 18% in KES; *P* < 0.001) decreased from baseline at 3 h in the eccentrically exercised leg, and remained below baseline at 48 and 72 h, with no significant group differences. Of eight measured plasma cytokines, interleukin‐6 (*P* = 0.008) and monocyte chemoattractant protein‐1 (*P* = 0.024) concentrations increased after 6 h, whereas tumour necrosis factor‐related apoptosis‐inducing ligand concentrations decreased after 3 h (*P* = 0.022) and 6 h (*P* = 0.011) post‐exercise with no significant group differences. Oral ketone monoester supplementation elevates plasma β‐OHB concentrations but does not prevent the decline in muscle force or alter plasma inflammatory cytokine profiles induced by eccentric exercise.

## 1 INTRODUCTION

Unaccustomed eccentric exercise causes skeletal muscle damage, which is characterized by a rapid and protracted decline in muscle force and a parallel increase in muscle soreness and acute inflammation (Jameson, Pavis, et al., 2021; Pavis et al., 2021). Successful remodelling of damaged muscle and recovery of muscle force likely depend on the capacity to elevate post‐exercise muscle protein synthesis rates to aid structural repair whilst limiting aberrant muscle protein breakdown in the face of acute inflammation (Jameson, Pavis, et al., 2021; Pavis et al., 2021). We recently reported that a dietary protein and polyphenol nutritional intervention approach that reduced acute intramuscular inflammation was associated with accelerated recovery of muscle force within 48 h following a single bout of eccentric exercise in healthy males and females (Jameson, Pavis, et al., 2021). Indeed, there is a strong evidence base demonstrating that acute nutritional supplementation strategies can enhance the recovery of muscle force with 72 h of intense exercise (e.g., Cooke et al., 2010, 2009; Draganidis et al., 2017; Howatson et al., 2010; Michailidis et al., 2013), although granted this may not be beneficial for long term adaptation (Owens et al., 2019).

β‐Hydroxybutyrate (β‐OHB) is a ketone body produced endogenously during carbohydrate deprivation, extended fasting or prolonged glycogen‐depleting exercise (Newman & Verdin, 2017). Elevations in circulating ketone bodies, via either increased endogenous production or exogenous intravenous infusion, have been associated with reduced muscle protein breakdown under various stress conditions including injury (Williamson et al., 1977), increased muscle protein synthesis signalling with protein ingestion following exercise (Vandoorne et al., 2017), energy restriction (Pawan & Semple, 1983) and inflammation (Thomsen et al., 2018). Indeed, intravenous administration of β‐OHB achieving plasma concentrations of 3.5 mM decreases forearm phenylalanine rate of appearance (a measure of muscle protein breakdown) by 70% during acute endotoxaemia, resulting in a more positive net muscle protein balance in healthy males (Thomsen et al., 2018). Moreover, elevations in β‐OHB are associated with a reduction in the activity of the NLRP3 inflammasome and downstream cytokine production in both cellular and animal models and in humans (Bae et al., 2016; Walsh et al., 2021; Youm et al., 2015). Increasing plasma β‐OHB nutritionally (in the absence of carbohydrate or energy restriction) may therefore be a novel and practical strategy to accelerate muscle recovery under inflammatory conditions.

We have previously demonstrated that circulating β‐OHB concentrations can be increased to ∼3 mM in healthy, rested, non‐energy restricted individuals via ingestion of the ketone monoester (*R*)‐3‐hydroxybutyl (*R*)‐3‐hydroxybutyrate, which is rapidly metabolized to the d‐isoform of β‐OHB (Myette‐Côté et al., 2018; Neudorf et al., 2019). However, whether elevated plasma β‐OHB via ketone ingestion can accelerate muscle force recovery and/or mitigate the systemic inflammatory response following muscle‐damaging eccentric exercise remains unknown. The present study employed eccentric exercise to induce acute inflammation and a decline in muscle force to test the hypothesis that increasing circulating β‐OHB via thrice daily oral ingestion of a ketone monoester drink could accelerate the recovery of muscle force in healthy, young volunteers undergoing conditions of full dietary control.

## 2 METHODS

### 2.1 Ethical approval

Participants were informed of the experimental procedures, potential risks, and the purpose of the study before providing full written and informed consent. The study was approved by the Sport and Health Sciences Ethics Committee of the University of Exeter (Ref. No. 109703/A/01). The study conformed to the standard set by the *Declaration of Helsinki*, except for registration in a database.

### 2.2 Participants

Sixteen young, healthy males (*n* = 6) and females (*n* = 10) (age = 23 ± 3 years, BMI = 22 ± 3 kg/m^2^) volunteered to take part in the present study. Participants attended the laboratory for a routine medical screening and completed a general medical questionnaire to assess their eligibility for participation. Exclusion criteria included a musculoskeletal injury, cigarette smoking, regular over‐the‐counter or prescribed medication use including non‐steroidal anti‐inflammatory medication, a diagnosed metabolic or cardiovascular impairment, routine use of nutritional supplements and a habitual protein intake of <0.8 g/kg BM/day assessed using a 3‐day diet diary. Inclusion criteria included being aged 18–40 years, a BMI of >18 and <30 kg/m^2^ and participating in recreational activity in the 6 months preceding the study (defined as exercise or sporting activities >2 h/week, but not structured exercise training such that participants were unaccustomed to maximal eccentric exercise). Female participants not using an oral contraceptive participated during days 6–12 of a regular menstrual cycle (i.e., mid‐follicular phase).

### 2.3 Experimental protocol

**FIGURE 1:**
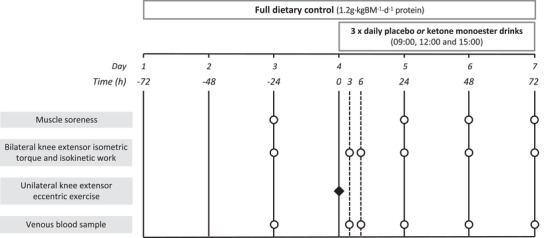
Schematic representation of the experimental protocol. Participants were provided with full dietary control (1.2 g/kg BM/day dietary protein) for 6 days. Unilateral eccentric knee extensor contractions were performed at ∼08.00 h on day 4 (time = 0 h). Venous blood was sampled, and knee extensor isometric torque and isokinetic function were measured at baseline (day 3) and 3, 6, 24, 48 and 72 h after eccentric contractions. Muscle soreness was measured at baseline and 24, 48 and 72 h after eccentric contractions. Participants ingested either ketone monoester (*n* = 8) or energy‐matched placebo (*n* = 8) drinks thrice daily on days 4, 5 and 6.

Following screening and acceptance into the study, all participants attended a familiarization visit to familiarize them with dynamometry and muscle soreness measurement protocols. Familiarization to the eccentric exercise protocol consisted of just five submaximal repetitions so as not to induce any repeated‐bout‐effect adaptations. At least 48 h following the familiarization visit, participants completed a 7‐day experimental period under fully controlled dietary conditions (i.e., all food provided with set macronutrient profile based on individualized energy balance – details below and experimental protocol shown in Figure 1). The first 3 days served as a run‐in period for dietary control with baseline muscle function testing performed on day 3. A unilateral bout of maximal eccentric muscle contractions of the knee extensors was performed using a Biodex System 3 isokinetic dynamometer (Biodex Medical Systems, Shirley, NY, USA) on the morning of day 4, randomly counterbalanced for leg dominance with the contralateral leg acting as a within‐participant control. Muscle soreness (measured via a visual analogue scale), and knee extensor peak isometric torque and total isokinetic work (measured using isokinetic dynamometry) were measured separately in both legs at baseline (day 3) and following 3 and 6 h recovery, then at 24, 48 and 72 h (i.e., the mornings of days 4–7) after eccentric contractions. In a parallel groups design, participants were block‐randomized using a computer‐generated sequence equally distributing leg dominance between groups to ingest either thrice daily ketone ester (KES; *n* = 8) or placebo (PLA; *n* = 8) drinks (see ‘Experimental drinks’) on the day of, and for 2 days after, eccentric contractions. Venous blood samples were also collected from the antecubital vein at baseline and 3, 6, 24, 48 and 72 h after eccentric contractions to measure the plasma concentration of β‐OHB and circulating cytokines. Body mass was measured overnight fasted on days 3, 4, 5, 6 and 7.

### 2.4 Eccentric knee extensor contractions

At ∼08.00 h on day 4 after a 10 h overnight fast, participants performed 300 (10 sets of 30 repetitions) voluntary maximal, unilateral, isokinetic, eccentric contractions of the knee extensors – a protocol we have previously demonstrated to impair muscle function and initiate inflammatory signalling in skeletal muscle within 24 h (Jameson, Kilroe, et al., 2021; Jameson, Pavis, et al., 2021; Pavis et al., 2021). Each contraction was performed at 60°/s over an 80° range of motion, which ended at full voluntary knee flexion. Each set was separated by 120 s of rest. Participants were instructed to resist the movement maximally throughout the full range of motion and verbal encouragement was provided throughout.

### 2.5 Muscle function testing

Muscle soreness and knee extensor peak isometric torque and total isokinetic work were determined as we have previously described (Jameson, Pavis, et al., 2021; Pavis et al., 2021) after a 10 h overnight fast at baseline and 24, 48 and 72 h after eccentric contractions. Additional measurements of knee extensor peak isometric torque and total isokinetic work were performed 3 and 6 h after eccentric contractions at which time participants were 2 h postprandial. Lower body muscle soreness was measured by asking participants to stand from a seated position and rate the corresponding sensation of lower body muscle soreness on a 100‐mm visual analogue scale anchored by ‘no pain’ at 0 mm and ‘worst possible pain’ at 100 mm. Knee extensor peak isometric torque was determined from three maximal isometric contractions at 75° of knee flexion (where 0° is equal to maximal voluntary knee extension) separated by 60 s of rest, after performing an incremental warm‐up of four submaximal contractions. Total isokinetic work was determined from the area under the torque–time curve after 30 maximal, concentric, isokinetic knee extensor contractions performed at 75°/s through an 80° range of motion equidistant from voluntary maximal knee extension and flexion and preceded by a five‐repetition submaximal warm‐up. All exercise was performed unilaterally, and verbal encouragement was provided throughout.

### 2.6 Dietary control

During the 7‐day experimental period, participants received a fully controlled diet from the research team with energy requirements calculated as the basal metabolic rate (estimated via the Henry equations) (Henry, 2005) multiplied by an activity factor of 1.6 in order to maintain energy balance, as evidenced in our previous work (Jameson, Pavis, et al., 2021; Pavis et al., 2022, 2021). Participants received all food products in individual packets and received step‐by‐step recipes. All meals and snacks were provided, whereas water and non‐caloric drinks were allowed ad libitum. Caffeinated drinks were only permitted after completion of each testing visit. Daily protein intake was standardized at 1.2 g/kg BM/day (∼17% of energy intake), with the remaining calories being contributed by fat (∼33% of energy intake) and carbohydrate (∼50% of energy intake). It has previously been demonstrated that administration of ketone monoester drinks with protein can potentiate muscle protein synthesis signalling pathways (Vandoorne et al., 2017). Thus, on day 4 participants consumed their controlled breakfast 1 h after eccentric exercise (providing 0.20 g/kg BM protein) and controlled lunch 1 h after muscle function testing at 3 h (providing 0.35 g/kg BM protein) to reduce the influence of nutrient distribution between individuals on acute recovery. Participants consumed their controlled diet ad libitum on days 1, 2, 3, 5 and 6. Compliance with the nutritional intervention was assessed via completed 6‐day food diaries, returned food containers and daily communication with the participants. Non‐compliance (i.e., consumption of food products not provided or failure to consume all products provided) was accounted for in the final dietary intake analysis.

### 2.7 Experimental drinks

Ketone and placebo drinks were taste‐, texture‐ and energy‐matched and prepared and coded by a member of research staff not associated with the study to ensure double blinding. Each ketone drink serving (150 ml total volume) was prepared by mixing 25 ml of ketone monoester (equating to 26.7 g of (*R*)‐3‐hydroxybutyl (*R*)‐3‐hydroxybutyrate, deltaG from TdeltaS Ltd, Thame, UK) with 120 ml water, 4 g of calorie free sweetener (Truvia, The Silver Spoon Company, Peterborough, UK) and 2 ml of calorie‐free blackcurrant flavouring (Robinsons Mini, Britvic Soft Drinks Ltd, Dublin, Ireland). Each placebo drink serving (150 ml total volume) was prepared by mixing 25 g of maltodextrin (Bulk Powders, Colchester, UK) with 120 ml water, 4 g of calorie free sweetener (Truvia), 2 ml of calorie‐free blackcurrant flavoring and 50 µl of Mavala Stop (Mavala, Geneva, Switzerland) to flavour‐match the bitterness of the ketone monoester.

Drinks were consumed immediately after eccentric contractions (∼09.00 h) and after functional testing at 3 h (∼12.00 h) and 6 h (∼15.00 h) post‐exercise on day 4, and at the same times of day on days 5 and 6 (i.e., nine drinks in total designed to elevate blood β‐OHB concentrations across waking hours of recovery within the limits of safety and tolerability of the ketone drink (Clarke et al, 2012). Participants were instructed not to consume food for 1 h following ketone monoester ingestion. Drinks were well tolerated, consumed within the allotted time (i.e., 5 min), and resulted in no reported adverse effects during or after the experimental period.

### 2.8 Blood sample collection and analysis

Ten millilitres of venous blood from the antecubital vein was collected at baseline and 3, 6, 24, 48 and 72 h after eccentric contractions. Venous blood was collected into lithium heparin‐containing tubes (BD vacutainer LH; BD Diagnostics, Nu‐Care, Stewartby, UK) and centrifuged immediately at 3000 *g* at 4°C for 10 min. Blood plasma was aliquoted and frozen at −80°C until subsequent analysis. For measurement of plasma β‐OHB, plasma samples were diluted (2‐fold and 10‐fold for PLA and KES, respectively) and analysed using a commercially available colorimetric enzyme assay according to the manufacturer's instructions (no. 700190, Cayman Chemical Co., Ann Arbor, MI, USA). All plates analysed for plasma β‐OHB had an average coefficient of variation (CV) below 5% based on duplicate samples. The plasma concentrations of interleukin (IL)‐1β, IL‐1ra, IL‐4, IL‐6, IL‐10, IL‐15, granulocyte colony stimulating factor (G‐CSF), monocyte chemoattractant protein‐1 (MCP‐1), interferon γ (IFNγ) and tumour necrosis factor‐related apoptosis‐inducing ligand (TRAIL) were measured in 2‐fold diluted blood plasma in duplicate using a multiplex assay according to the manufacturer's instructions using manufacturer‐supplied reagents, antibodies and the MESO QuickPlex SQ 120 instrument (U‐PLEX, Meso Scale Diagnostics LLC (MSD), Rockville, MD, USA). All plates analysed for circulating cytokines had an average CV below 6% based on duplicate samples.

### 2.9 Statistics

In line with our previous work investigating the recovery of muscle soreness and function after eccentric contractions (Jameson, Pavis, et al., 2021; Pavis et al., 2021), isometric torque and isokinetic work in the eccentrically exercised leg were corrected to the contralateral control leg prior to statistical analysis to reduce inter‐ and intra‐individual variation. Repeated measures two‐factor analysis of variance (ANOVA) tests (with group as a between‐participant factor, and time as a within‐participant factor) were used to compare differences in muscle soreness, isometric torque, isokinetic work, body mass and circulating β‐OHB and cytokine concentrations. For all ANOVAs, when significant main and/or an interaction effect were found, Šidák's *post hoc* test was applied for pairwise comparisons. Data are expressed as means ± standard deviations (SD). Statistical significance was set at *P* < 0.05 and statistical analyses were performed with GraphPad Prism version 9.0.1(GraphPad Software, San Diego, CA, USA).

## 3 RESULTS

### 3.1 Participant characteristics and diet

**FIGURE 2:**
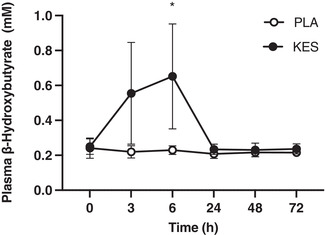
Plasma β‐hydroxybutyrate measured in venous blood samples collected before (0 h) and 3, 6, 24, 48 and 72 h following 300 unilateral eccentric knee extensor contractions. Ketone monoester (*n* = 8; filled circles; KES) or isocaloric maltodextrin placebo (*n* = 8; open circles; PLA) drinks were ingested immediately, 3 and 6 h after eccentric exercise and at the same times of day during the 2 days following eccentric exercise. Data are presented as means with error bars representing standard deviation. Statistical analysis was performed with a 2‐factor analysis of variance. *Plasma β‐hydroxybutyrate greater in KES compared with PLA, *P* < 0.05.

Participants’ age (PLA 23 ± 3, KES 23 ± 3 years; *P* = 0.873), body mass (PLA 63 ± 11, KES 66 ± 10 kg; *P* = 0.563), height (PLA 168 ± 8, KES 170 ± 10 cm; *P* = 0.612) and BMI (PLA 22 ± 3, KES 23 ± 3 kg/m^2^) were not different between groups. Average daily energy and macronutrient ingestion during the 6‐day controlled diet after accounting for compliance was 10.2 ± 0.1 MJ with 52 ± 3% energy as carbohydrate, 31 ± 3% energy as fat, and 13 ± 1% energy (1.2 ± 0 g/kg BM/day) as protein. Body mass did not change during the experimental period (*P* = 0.708) and was not different between groups (*P* = 0.426). Baseline plasma β‐OHB concentrations were equivalent between groups: 0.24 ± 0.06 mM in PLA and 0.25 ± 0.03 mM in KES (*P* = 0.750). Plasma β‐OHB demonstrated a significant effect of group (*P* = 0.0003) such that β‐OHB was greater in KES compared with PLA at 3 h (0.56 ± 0.13 *vs*. 0.22 ± 0.04 mM, respectively; *P* = 0.0798) and 6 h (0.65 ± 0.41 *vs*. 0.23 ± 0.02 mM, respectively; *P* = 0.0312; Figure 2).

### 3.2 Muscle soreness, total isokinetic work and peak isometric torque

**FIGURE 3:**
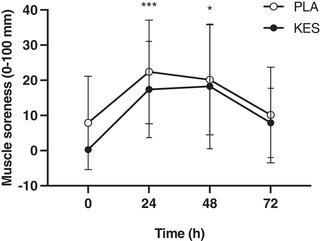
Lower body muscle soreness measured on a 100 mm visual analogue scale with 0 mm representing ‘no pain’ and 100 mm representing ‘worst possible pain’. Measurements were taken before (0 h) and 24, 48 and 72 h following 300 unilateral eccentric knee extensor contractions. Ketone monoester (*n* = 8; filled circles; KES) or isocaloric maltodextrin placebo (*n* = 8; open circles; PLA) drinks were ingested immediately, 3 and 6 h after eccentric exercise and at the same times of day during the 2 days following eccentric exercise. Data are presented as means with error bars representing standard deviation. Statistical analysis was performed with a 2‐factor analysis of variance. Main effect of time: *P < 0.05, **P < 0.01.

Muscle soreness was not different between groups at baseline (*P* = 0.126) and increased from baseline in both groups after 24 h (from 8 ± 13 to 22 ± 15 mm in PLA and from 0 ± 0 to 17 ± 14 mm in KES; *P* = 0.000487), remained above baseline at 48 h (20 ± 6 mm in PLA and 18 ± 6 mm in KES; *P* = 0.0154) and was no longer different from baseline after 72 h in either group (*P* = 0.449; Figure 3) with the increase in muscle soreness not differing between groups (*P* = 0.747).

**FIGURE 4:**
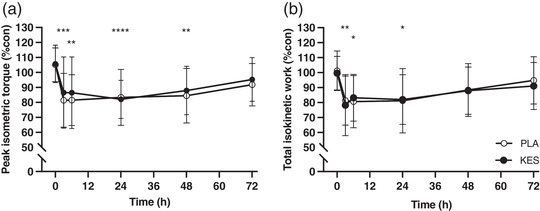
Knee extensor peak isometric torque (a) and total isokinetic work (b) expressed relative to the contralateral control leg. Measurements were taken before (0 h) and 3, 6, 24, 48 and 72 h following 300 unilateral eccentric knee extensor contractions. Ketone monoester (*n* = 8; filled circles; KES) or isocaloric maltodextrin placebo (*n* = 8; open circles; PLA) drinks were ingested immediately, 3 and 6 h after eccentric exercise and at the same times of day during the 2 days following eccentric exercise. Data are presented as means with error bars representing standard deviation. Statistical analysis was performed with separate 2‐factor analysis of variance. Main effect of time: *P < 0.05, **P < 0.01, ***P < 0.001, ****P < 0.0001.

Peak isometric torque decreased from baseline after 3 h (by 23 ± 13% in PLA and 20 ± 18% in KES; *P* = 0.000612), remained below baseline at 6 h (by 22 ± 13% in PLA and 21 ± 20% in KES; *P* = 0.00122), 24 h (by 22 ± 14% in PLA and 23 ± 11% in KES; *P* < 0.0001) and 48 h (by 20 ± 15% in PLA and 18 ± 15% in KES; *P* = 0.00101) and was no longer different from baseline after 72 h in either group (*P =* 0.0610; Figure 4a). Total isokinetic work decreased from baseline after 3 h (by 20 ± 21% in PLA and 21 ± 19% in KES; *P* = 0.00827), remained below baseline at 6 h (by 21 ± 20% in PLA and 16 ± 19% in KES; *P* = 0.0146) and 24 h (by 20 ± 22% in PLA and 18 ± 17% in KES; *P* = 0.0126) and was no longer different from baseline after 48 h in either group (*P* = 0.175; Figure 4b). The decreases in peak isometric torque (*P* = 0.933) and total isokinetic work (*P* = 0.942) were not different between groups.

### 3.3 Plasma cytokine concentrations

**FIGURE 5:**
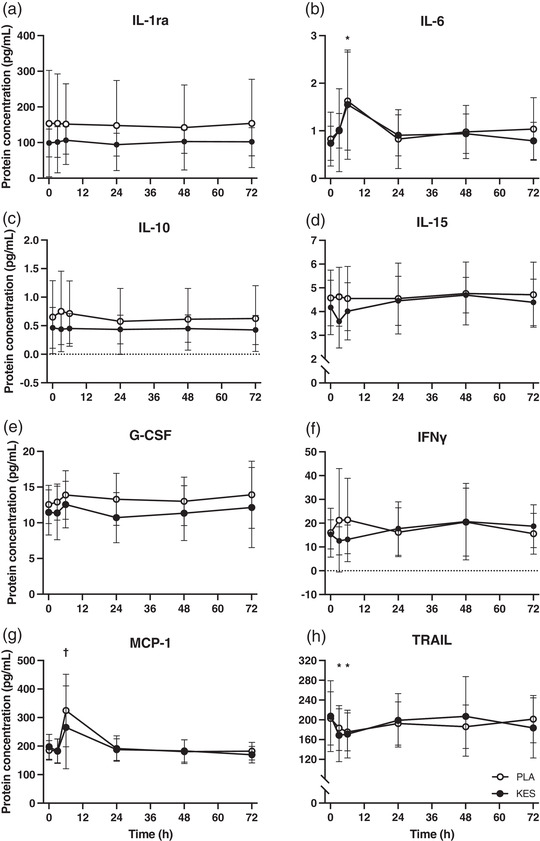
Plasma cytokine concentrations measured in venous blood samples collected before (0 h) and 3, 6, 24, 48 and 72 h following 300 unilateral eccentric knee extensor contractions. Ketone monoester (*n* = 8; filled circles; KES) or isocaloric maltodextrin placebo (*n* = 8; open circles; PLA) drinks were ingested immediately, 3 and 6 h after eccentric exercise and at the same times of day during the 2 days following eccentric exercise. Data are presented as means with error bars representing standard deviation. Statistical analysis was performed with separate 2‐factor analysis of variance. *Time point different from 0 h; †time point different from 3 h; *P* < 0.05.

The plasma concentrations of IL‐1β and IL‐4 were below the limits of detection and so are excluded from analysis. The plasma concentration of IL‐6 (*P* = 0.000876), MCP‐1 (*P* = 0.00171) and TRAIL (*P* = 0.00515) demonstrated main effects of time (Figure 5). *Post hoc* tests identified that IL‐6 concentrations increased between baseline and 6 h (from 0.83 ± 0.57 to 1.63 ± 1.03 pg/ml in PLA and from 0.74 ± 0.36 to 1.55 ± 1.14 pg/ml in KES, respectively; *P* = 0.00795) and MCP‐1 concentrations increased between 3 and 6 h (from 182.2 ± 41.8 to 324.9 ± 1127.1 pg/ml in PLA and from 182.1 ± 42.6 to 265.8 ± 145.3 pg/ml in KES, respectively; *P* = 0.0244). The plasma concentrations of TRAIL decreased between baseline and 3 h (from 202.8 ± 53.8 to 183.5 ± 45.6 pg/ml in PLA and from 207.5 ± 71.6 to 168.9 ± 53.5 pg/ml in KES, respectively; *P* = 0.0219) and baseline and 6 h (to 175.6 ± 38.3 pg/ml in PLA and to 171.2 ± 48.3 pg/ml in KES; *P* = 0.0113). Changes in IL‐6 (*P* = 0.927), MCP‐1 (*P* = 0.502) and TRAIL (*P* = 0.165) concentrations were not different between PLA and KES groups. No significant differences were noted in IL‐1ra, IL‐10, IL‐15, G‐CSF or IFNγ (Table 1).

**TABLE 1:** Plasma cytokine concentrations

	PLA						KES						*P*		
	0 h	3 h	6 h	24 h	48 h	72 h	0 h	3 h	6 h	24 h	48 h	72 h	Time	Group	Interaction
IL1‐RA	153.4 ± 149.2	153.9 ± 138.7	151.7 ± 113.2	147.9 ± 126.4	142.3 ± 119.1	153.7 ± 123.7	98.8 ± 39.0	101.6 ± 43.0	106.6 ± 39.0	94.1 ± 32.1	102.9 ± 33.0	102.3 ± 39.5	0.456	0.332	0.629
IL‐6	0.8 ± 0.6	1.0 ± 0.4	1.6 ± 1.0	0.8 ± 0.6	1.0 ± 0.6	1.0 ± 0.7	0.7 ± 0.4	1.0 ± 0.9	1.6 ± 1.2	0.9 ± 0.4	0.9 ± 0.4	0.8 ± 0.4	0.001	0.830	0.927
IL‐10	0.7 ± 0.6	0.8 ± 0.7	0.7 ± 0.6	0.6 ± 0.6	0.6 ± 0.5	0.6 ± 0.6	0.5 ± 0.4	0.4 ± 0.3	0.5 ± 0.3	0.4 ± 0.3	0.5 ± 0.3	0.4 ± 0.3	0.314	0.369	0.394
IL‐15	4.6 ± 1.2	4.6 ± 1.2	4.6 ± 1.4	4.6 ± 1.5	4.8 ± 1.3	4.7 ± 1.3	4.2 ± 1.2	3.6 ± 1.1	4.0 ± 1.2	4.5 ± 1.0	4.7 ± 0.7	4.4 ± 1.0	0.096	0.458	0.191
G‐CSF	12.6 ± 2.7	12.9 ± 2.5	13.9 ± 3.4	13.3 ± 3.6	13.0 ± 3.4	13.9 ± 4.7	11.5 ± 3.2	11.4 ± 3.8	12.6 ± 3.3	10.7 ± 3.5	11.4 ± 3.8	12.1 ± 5.6	0.340	0.316	0.943
IFNγ	16.0 ± 10.3	21.2 ± 21.7	21.4 ± 17.5	16.2 ± 10.3	20.5 ± 14.3	15.6 ± 8.6	15.3 ± 6.1	12.6 ± 5.9	13.2 ± 6.0	17.7 ± 11.2	20.7 ± 16.1	18.7 ± 9.0	0.559	0.665	0.252
MCP‐1	185.2 ± 34.2	183.2 ± 41.8	324.8 ± 127.1	191.4 ± 44.5	180.4 ± 41.6	182.3 ± 30.9	197.5 ± 43.7	182.1 ± 42.6	265.7 ± 145.3	187.6 ± 37.9	183.1 ± 39.1	169.9 ± 29.0	0.002	0.650	0.502
TRAIL	202.8 ± 53.8	183.4 ± 45.5	175.6 ± 38.3	192.6 ± 43.8	186.0 ± 44.0	201.4 ± 47.8	207.5 ± 71.6	168.9 ± 53.5	171.2 ± 48.3	199.1 ± 54.1	207.0 ± 80.5	183.6 ± 60.8	0.005	0.976	0.165

Values represent means ± SD. h, hours after 300 unilateral eccentric quadriceps contractions (0 h was sampled before exercise). Analysis was performed using repeated measure 2 factor ANOVAs. Abbreviations: G‐CSF, granulocyte colony stimulating factor; IFNγ, interferon γ; IL, interleukin; IL1‐RA, IL‐1 receptor antagonist; KES, ketone monoester; MCP‐1, monocyte chemoattractant protein 1; PLA, placebo; TRAIL, TNF‐related apoptosis‐inducing ligand.

## 4 DISCUSSION

An anti‐catabolic role of β‐OHB has been reported in catabolic stress conditions such as injury (Williamson et al., 1977), energy restriction (Nair et al., 1988) and acute inflammation (Thomsen et al., 2018). In the present study we initiated an acute inflammatory response and impairment to muscle function using muscle‐damaging eccentric exercise and investigated whether increasing plasma β‐OHB concentrations using oral ketone monoester supplementation would modulate these responses. Our findings in healthy males and females undergoing a strict dietary control show that oral ketone monoester supplementation did not prevent the decline in muscle force caused by eccentric exercise, nor impact circulating concentrations of select inflammatory cytokines.

A single bout of 300 eccentric knee extensor contractions causes a transient decline in muscle force (Draganidis et al., 2016; Jameson, Pavis, et al., 2021; Michailidis et al., 2013; Paulsen et al., 2010; Pavis et al., 2021) which correlates with ultrastructural myofibrillar disruption (Raastad et al., 2010). In the present study we used the established unilateral within‐participant damaged versus control leg approach (Jameson, Pavis, et al., 2021; Pavis et al., 2021) and report relative to the contralateral control leg an immediate (3 h) decline in isometric torque and isokinetic work of ∼25% and ∼20%, respectively. Characteristically of muscle damage, muscle force did not appreciably begin to recover until at least 48 h post‐exercise (PLA group; Figure 4). Additionally, the delayed increase in muscle soreness further supports the presence of muscle (Draganidis et al., 2017; Michailidis et al., 2013; Pavis et al., 2021) (Figure 3). Consistent with the well characterized acute cytokine‐mediated inflammatory response to muscle damage (McKay et al., 2009; Paulsen et al., 2005), we observed an approximate two‐fold increase in plasma IL‐6 and MCP‐1 concentrations 6 h post‐exercise (PLA group; Figure 5b, g), which largely preceded and may therefore contribute to the protracted loss of force. To our knowledge the present study is the first to measure TRAIL concentrations after eccentric exercise, which decreased at 3 and 6 h (Figure 5h). This is consistent with our previous work showing accelerated recovery of muscle force after eccentric exercise occurred in parallel with attenuated muscle mRNA expression of the TRAIL receptor TNF receptor superfamily member 10B (Jameson, Pavis, et al., 2021), highlighting TRAIL signalling as a possible novel mediator of muscle recovery.

Plasma β‐OHB concentrations were elevated ∼3‐fold above fasting (to ∼0.6 mM) 3 h after ketone monoester drink consumption (Figure 2, time points 3 and 6 h). This increase is consistent with expectations based on our previous time course work where we report plasma β‐OHB concentrations of ∼1.2 mM 2 h post‐ingestion of a similar ketone monoester dose (Myette‐Côté et al., 2018; Neudorf et al., 2019). Therefore, we can state with confidence that plasma β‐OHB concentrations were likely elevated above fasting for at least 9 h of waking recovery from eccentric exercise (i.e., for at least 3 h after each of the thrice daily drinks which were consumed 3 h apart). Based on our and others (Clarke et al., 2012) previous time course work, we would expect plasma β‐OHB concentrations to have reached 3 mM within 30 min of ingestion. However, our blood sampling time points were not designed to capture peak plasma β‐OHB concentration, and the exercise protocol employed in the present study may have increased plasma β‐OHB clearance, so this may not have been the case. Nevertheless, this dosing regimen ensured β‐OHB was elevated when plasma cytokine concentrations were greatest (Figure 5) and is comparable to the work of Thomsen et al. who reported that plasma β‐OHB concentrations of 3.5 mM decreased forearm muscle protein breakdown (phenylalanine rate of appearance) during acute endotoxaemia (Thomsen et al., 2018). The reason for a lack of effect of β‐OHB on the recovery of muscle force in the present study is not clear, but it could be that our ketone dosing strategy was insufficient given that previous studies demonstrating an effect on muscle protein metabolism have used higher doses and achieved plasma β‐OHB concentrations in excess of 3 mM (Thomsen et al., 2018; Vandoorne et al., 2017). Indeed, such doses affected leucine metabolism in particular (Vandoorne et al., 2017), which is thought to be essential for stimulating muscle protein synthesis, a prerequisite for recovery. Thus, whilst we controlled for dietary protein and leucine intake, a limitation of the present study was that muscle protein synthesis or leucine signalling was not measured in the present study as we have done in the past (Jameson et al., 2021; Pavis et al., 2021). Moreover, the magnitude or context of inflammation caused by eccentric exercise versus endotoxaemia may be insufficient. Indeed the reduction in forearm muscle protein breakdown reported by Thomsen et al. during β‐OHB and endotoxin infusion was accompanied by fever and markedly greater peak plasma IL‐6 (3193 pg/ml) and IL‐1β concentrations (Thomsen et al., 2018) whereas we observed peak IL‐6 concentrations of just 1.6 pg/ml after eccentric exercise (Figure 5b) and IL‐1β was below the limit of detection, consistent with other studies that have failed to measure systemic changes in IL‐1β after eccentric exercise (Cornish & Johnson, 2014; Hirose et al., 2004). Moreover, the ∼25% reduction in muscle force observed in the present study was less than the ∼35–40% reduction in muscle force reported elsewhere following similar eccentric exercise protocols (Jameson, Pavis, et al., 2021; Paulsen et al., 2010; Pavis et al., 2021), perhaps because muscle function testing was performed 3 and 6 h after eccentric exercise, which we speculate would stimulate an increase in protein turnover that would help to promote muscle recovery (Jameson, Pavis, et al., 2021; Pavis et al., 2021). β‐OHB did not suppress the increase in plasma IL‐6 and MCP‐1 measured 3–6 h post‐exercise (Figure 5b, g) suggesting that β‐OHB does not influence the acute systemic inflammatory response caused by eccentric muscle‐damaging exercise.

We employed a rigorous dietary control and standardized protein intake to mitigate any effect differences in energy or macronutrient availability could have on plasma β‐OHB within and between groups and the beneficial effect dietary protein intake can have on recovery from eccentric exercise (Buckley et al., 2010; Cockburn et al., 2008; Cooke et al., 2010; Draganidis et al., 2017; Jameson, Pavis, et al., 2021; Pavis et al., 2021). Furthermore, we chose to dose ketone monoester supplements thrice daily (as opposed to as a single post‐exercise dose) to elevate plasma β‐OHB for a sustained period of waking recovery and to complement the continual remodelling process that occurs for several days after eccentric exercise. There are, however, also some limitations to acknowledge. Firstly, we did not measure the time course of plasma β‐OHB concentrations after ketone monoester supplementation. Therefore, the exact peak plasma β‐OHB concentrations achieved in this participant cohort are not clear and can only be inferred based on our previous work in a different cohort supplementing with a similar ketone monoester dose (Myette‐Côté et al., 2018). Moreover, whilst we analysed the plasma concentrations of a broad range of both pro‐ and anti‐inflammatory cytokines, we did not investigate if ketone monoester supplementation modulated local inflammatory pathways that are known to be upregulated by eccentric exercise in skeletal muscle and mononuclear cells (García‐López et al., 2007; Jameson, Pavis, et al., 2021). Indeed, we have shown that ketone monoester supplementation in individuals with obesity reduces lipopolysaccharide‐stimulated monocyte caspase‐1 activation without modulating systemic cytokine concentrations (Walsh et al., 2021). The insulinotropic effect of energy‐matched placebo (25 g carbohydrate) and ketone monoester (26.7 g of ketone monoester) drinks was not measured and as such we cannot exclude an effect of insulin on post‐eccentric exercise muscle protein turnover (Abdulla et al., 2016). We included both males and females in this study, with control for menstrual cycle phase, but the study was not powered to test for potential sex differences. Future mechanistic human studies in both males and females are required to ascertain the effect of ketone monoester supplementation on local inflammatory pathways.

In conclusion, oral ketone monoester supplementation in healthy males and females undergoing a strict dietary control resulted in sustained increase in plasma β‐OHB but did not accelerate the recovery of muscle force in skeletal muscle damaged by eccentric exercise. Further, we report no impact of ketone monoester supplementation on plasma inflammatory cytokine profiles after eccentric exercise, but present novel data to demonstrate that a reduction in TRAIL signalling may be important in the remodelling of damaged skeletal muscle.

## AUTHOR CONTRIBUTIONS

All experiments were performed in the Nutritional Physiology Research Unit at the University of Exeter, UK. Tom S. O. Jameson, Benjamin T. Wall, Jonathan P. Little and Francis B. Stephens designed the experiment, Tom S. O. Jameson and Hashim Islam performed the experiment. Tom S. O. Jameson, Hashim Islam, Benjamin T. Wall, Jonathan P. Little and Francis B. Stephens analysed data. Tom S. O. Jameson and Francis B. Stephens drafted the manuscript and Hashim Islam, Benjamin T. Wall and Jonathan P. Little edited and revised the manuscript. Tom S. O. Jameson, Hashim Islam, Benjamin T. Wall, Jonathan P. Little and Francis B. Stephens approved the final version of the manuscript and agree to be accountable for all aspects of the work in ensuring that questions related to the accuracy or integrity of any part of the work are appropriately investigated and resolved. All persons designated as authors qualify for authorship, and all those who qualify for authorship are listed.

## CONFLICT OF INTEREST

None.

## Supporting information

Statistical Summary Document

## Data Availability

All data supporting the results of this publication are presented within it. Raw data generated during the current study are not publicly available but are available from the corresponding author on reasonable request.
